# Primary Arterial Hypertension Associated with Cognitive Dysfunction in Young Adults: Results from a Cross-Sectional Controlled Study

**DOI:** 10.3390/bs14040321

**Published:** 2024-04-12

**Authors:** Kristijonas Puteikis, Karolis Ažukaitis, Danguolė Dadurkevičienė, Kazys Simanauskas, Vaida Šileikienė, Augustina Jankauskienė, Rūta Mameniškienė

**Affiliations:** 1 Faculty of Medicine, Vilnius University, 03101 Vilnius, Lithuania; 2 Vilnius University Hospital Santaros Klinikos, 08406 Vilnius, Lithuania

**Keywords:** attention, cardiovascular risk, cognition, memory, neuropsychology, healthy aging

## Abstract

Despite evidence of primary hypertension (PH)-associated cognitive dysfunction in pediatric, middle-aged, and older adult populations, respective data in young adults remains scarce. We aimed to define differences in cognitive performance between individuals with PH and healthy controls in early adulthood. A convenience sample of young adults (age 18–45 years) with PH and their healthy sex, age, education, and household income matched counterparts were cross-sectionally tested for verbal fluency, verbal memory, general intelligence, reaction speed, attention, visual memory, and executive functioning. Between-group differences were determined using Student’s t and Mann-Whitney U tests. Sensitivity analysis was performed by adjusting for body mass index (BMI) in analysis of covariance (ANCOVA) and regression models. Thirty-three adults with PH (22, 66.7% male, median age 38.8, interquartile range (IQR) = 33.2–41.6 years) and twenty-two healthy controls (9, 40.9% male, median age 36.1, IQR = 27.5–39.8 years) completed the neuropsychological assessment. Participants with PH performed worse on computerized tasks of reaction time (median response time (Z = −2.019, *p* = 0.044), median time for release of response button (Z = −2.509, *p* = 0.012)) and sustained attention (signal detection measure, RVPA (t = 2.373, *p* = 0.021), false alarms ÷ (false alarms + correct rejections), RVPPFA (Z = −2.052, *p* = 0.040)). The group variable was not a statistically significant predictor of performance in these domains after adjustment for BMI (*p* > 0.05). In regression analyses, high office systolic blood pressure (oSBP) was independently associated with poor sustained attention (β_SBP(st.)_ = −0.283, multiple R^2^ = 0.252 (RVPA), β_SBP(st.)_ = 0.551, multiple R^2^ = 0.386 (RVPPFA)). Young adults with PH were found to perform worse in tasks of response speed and sustained attention. While the difference between neuropsychological evaluation results in PH and control groups was confounded by BMI, oSBP measures were independently related to sustained attention. The selectivity of PH-associated cognitive profile in young adults has to be confirmed in larger trials.

## 1. Introduction

With the increasing global burden of disorders of the nervous system, the value, promotion, and protection of brain health has become a major focus point in the World Health Organization (WHO)’s 2022–2031 intersectoral global action plan for epilepsy and other neurological disorders [1]. Brain health is defined by the WHO as “the state of brain functioning across cognitive, sensory, social–emotional, behavioral, and motor domains, allowing a person to realize their full potential over the life course, irrespective of the presence or absence of disorders”, and is viewed as an important mediator of other health and societal outcomes [2]. Therefore, the prevention and management of any medical and behavioral conditions leading to brain disorders are highly encouraged. Primary arterial hypertension (PH) is amongst the established modifiable risk factors for different burdensome neurological conditions, such as stroke or dementia [3]. It is suggested that hypertension-induced white matter damage, micro- and larger infarcts and microhemorrhages, as well as brain atrophy, lead to earlier onset of cognitive ageing and increase the likelihood of dementia [4,5,6]. As the clinical onset of stroke and dementia is usually late in life, the impact of PH on brain functioning has mostly been explored in older adult populations [7,8]. Further, there is ample evidence that the presence of hypertension in middle adulthood (age of around 40–64 years) is related to a higher probability of dementia onset later in life [8]. However, studies investigating hypertension-associated cognitive outcomes across early and middle adulthood remain scarce [9]. Results of the Coronary Artery Risk Development in Young Adults (CARDIA) Study [10,11] and the Young Finns Study [12] indicate that even very early exposure to arterial hypertension is associated with a worse cognitive profile among middle-aged adults. While such individuals have only subclinical levels of cognitive impairment, it may nonetheless be important for their health and societal functioning in later years. To better understand the influence of PH on brain health across the life spectrum, we believe it is essential to further explore the relationship between hypertension and cognitive outcomes in younger patients with PH, who are traditionally expected to have clinically normal brain function. In the current study, we aimed to comprehensively compare cognitive performance between adults aged 18–45 years with and without PH across both verbal and non-verbal cognitive domains. Our hypotheses were that individuals with PH would present with worse results in most cognitive tasks if compared to healthy counterparts, and that blood pressure measures would be inversely associated with cognitive performance.

## 2. Materials and Methods

### 2.1. Study Setting and Participants

This study was of cross-sectional design and was conducted between June 2021 and June 2023 at Vilnius University Hospital Santaros Klinikos, a tertiary medical institution in an urban area. A convenience sample was formed from young adults with primary arterial hypertension and healthy controls that had been consecutively invited to participate in the study during routine medical visits to the Centre for Family Medicine at the same hospital. We sought to recruit healthy participants with a similar distribution of sex, age, household income, and proportion of higher education. All participants were evaluated for blood pressure and cognitive outcomes at a single time point during the study.

Participant inclusion criteria were: (i) young adults (age 18–45 years [13]), (ii) Lithuanian speakers, and (iii) treated or non-treated primary arterial hypertension confirmed as defined by the European Society of Cardiology (ESC) and the European Society of Hypertension (ESH) guidelines of 2018 [14] (hypertension group only).

Participant exclusion criteria were: (i) significant sensory or motor deficits preventing completion of cognitive evaluation, (ii) cardiovascular comorbidities other than primary arterial hypertension (e.g., congenital heart disease causing hypoxia or hypoperfusion, hemodynamically significant heart rhythm or conduction disorders), (iii) diabetes mellitus, (iv) body mass index (BMI) >35 kg/m^2^, and (v) glomerular filtration rate <60 mL/min/1.73 m^2^ upon enrolment.

### 2.2. Anthropometric and Blood Pressure Measurement

Participants were measured for weight, height, and waist and hip circumference according to World Health Organization guidelines [15], and underwent office BP (oBP, Omron 705IT (HEM-759P-E2), ABPM (Mobile-O-Graph monitor) and central BP (SphygmoCor-XCEL) measurements according to ESC/ESH guidelines [14]. Mean systolic (SBP) and diastolic (DBP) office BP values were calculated by averaging the second and third measurements.

### 2.3. Cognitive Outcomes

Participants completed a battery of neuropsychological tests combining paper-pencil and computerized (Cambridge Neuropsychological Test Automated Battery (CANTAB) by Cambridge Cognition, Ltd., Cambridge, UK) assessments.

The battery included:

Categorical and phonemic verbal fluency (scored as number of nouns pronounced in a category and starting with the same letter, respectively, in one minute).

Verbal-logical story recall (VLS, verbal memory). Participants were read a short verbal story and had to recite it immediately, after 30 min and after 24 h. The story was scored on a 24-point scale for each item freely recalled.

Intelligence quotient (IQ) measured by the Wechsler Abbreviated Scale of Intelligence-II.

Non-verbal CANTAB tests (name of the test, abbreviation, and cognitive domain tested; detailed descriptions of each test are presented in Appendix A, Table A1):

- Match to Sample Visual Search (MTS, attention and processing speed)
- Paired Associates Learning (PAL, visual memory and new learning)
- Reaction Time Task (RTI, motor and mental response speed, movement time, reaction time, response accuracy and impulsivity)
- Rapid Visual Information Processing (RVP, sustained attention)
- Stockings of Cambridge (SOC, spatial planning, working memory)
- Spatial Span (SSP, working memory capacity)
- Spatial Working Memory (SWM, working memory, strategy use)

All tasks were performed under the guidance of a licensed clinical psychologist in a single session before ABPM measurement, except for IQ testing and 24 h VLS testing, which were completed upon taking off the ABPM cuff on the second day. CANTAB tests were performed in accordance with guidelines supplied by Cambridge Cognition, Ltd.

### 2.4. Statistical Analysis

The normality of continuous variables was assessed by means of the Shapiro-Wilk test. The results of anthropometric, cardiovascular, and cognitive evaluations were compared between the PH and control groups using the Student’s *t* (normal distribution) or Mann-Whitney U (non-normal distribution, scale variable) tests. The statistical significance of the Student’s *t* test was determined using the *t* value as a measure of difference between the mean values of selected variables in the PH and control groups. For the Mann-Whitney U test, the Z value was presented as an estimate of difference between the sum of ranked group data. Both *t* and Z values were used to yield a *p* value that either confirmed or rejected the hypothesis that there was no statistically significant difference in data distribution between the PH and control groups. The distribution of categorical variables was compared using the Chi-squared (χ^2^) test. Verbal memory decay between groups was compared by means of a two-way repeated measures analysis of variance (ANOVA) with three time points (immediate, 30 min and 24 h recall of the verbal-logical story) as the within-group variable and as a between-group (PH or control group) differentiation. Post-hoc Bonferroni-adjusted simple effects analysis was performed to compare between-group performance at each of the three points in time. Correlation coefficients (Pearson’s r) were calculated in search for association between cognitive outcomes, BP, and BMI.

Sensitivity analysis was performed for the results of cognitive testing if the difference between groups was statistically significant. The respective cognitive variables were included as dependent factors in ANCOVA models adjusted for BMI. Maximum likelihood regression modelling analysis was performed using the IBM SPSS Statistics 22 software for mediation models “AMOS”, with cognitive task results found to be correlated with both BMI and BP treated as dependent variables. The direct association of both BMI and BP with cognitive variables was estimated (i.e., neither of the two independent variables was treated as a mediator); therefore, this method was comparable to simple regression modelling rather than mediation, which yields both direct and indirect effects on the dependent variable.

All statistical tests were two-tailed, and the level of significance was set at *p* < 0.05. Because of the exploratory nature of the study, statistical analysis was not adjusted for multiple testing. All analyses were conducted in IBM SPSS v26.

The target sample size of the study that was deemed feasible to achieve was *n* = 52, allowing detection of a difference of 12 IQ points (effect size d = 0.8) with β = 0.8 and α = 0.05 (G*Power 3.1.9.7).

### 2.5. Ethics

The study was approved by the Vilnius Regional Biomedical Research Ethics Committee (approval no. 2021/5-1348-821) and conducted in accordance with the principles of the World Medical Association Helsinki declaration as well as local law. All participants provided written informed consent upon enrolment.

## 3. Results

The study sample consisted of 33 adults with PH (22, 66.7% male) and 22 healthy controls (9, 40.9% male), matched for sex (χ^2^ = 3.561, *p* = 0.059), age (Z = −1.581, *p* = 0.114), household income (Z = −0.850, *p* = 0.395), and education (81.8% vs. 95.2% with higher education, *p* = 0.227). The anthropometric and cardiovascular characteristics of the study sample are presented in Table 1. Nineteen (57.6%) participants with PH had been treated for the condition with antihypertensive drugs of either one (8, 42.1%), two (8, 42.1%), three (2, 10.5%), or five (1, 5.3%) different categories. Five (15.2%) individuals with PH (all treated) had normal BP measurements across office BP and ABPM measures during the study.

Participants with PH performed worse on computerized tasks of reaction time (RTI) and sustained attention (RVP), as seen in Table 2. The difference was no longer statistically significant after adjusting for BMI (BMI F(1, 51) = 9.416, *p* = 0.003, group F(1, 51) = 0.524, *p* = 0.472 [RTIFMDMT], BMI F(1, 51) = 0.003, *p* = 0.953, group F(1, 51) = 2.648, *p* = 0.110 [RTISMDRT], BMI F(1, 51) = 6.699, *p* = 0.013, group F(1, 51) = 0.189, *p* = 0.666, [RVPA], BMI F(1, 51) = 6.196, *p* = 0.016, group F(1, 51) = 0.005, *p* = 0.945 [RVPPFA]).

**Figure 1:**
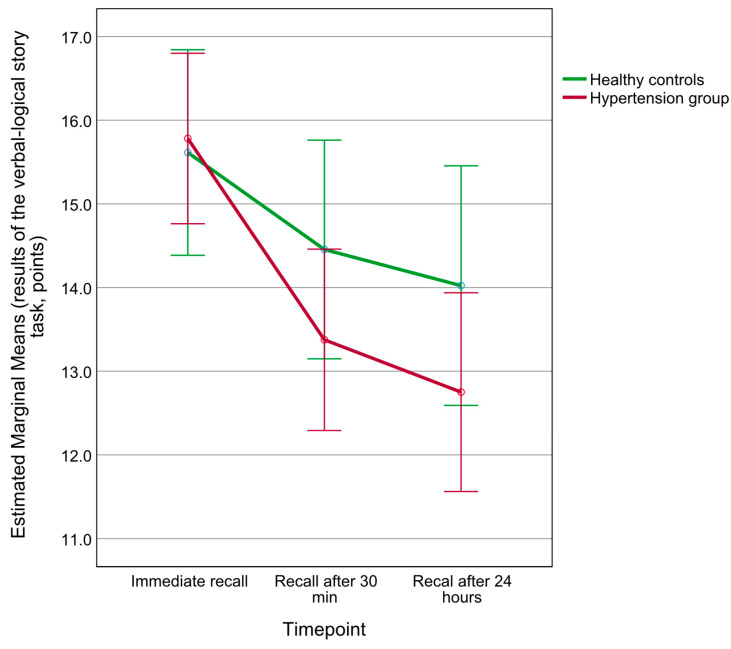
Comparison of performance between participants with PH and healthy controls in the task of verbal-logical story recall at three delays. Error bars represent 95% confidence intervals.

While participants with PH and healthy controls had similar recall scores on the verbal-logical story task, a significant delay by group interaction was detected in the two-way repeated measures ANOVA model (delay effect F(1.7, 88.0) = 44.113, *p* < 0.001, $\eta_{p}^{2}$ = 0.46, group effect F(1, 52) = 0.819, *p* = 0.370, $\eta_{p}^{2}$ = 0.02, delay by group effect F(1.7, 88.0) = 4.597, *p* = 0.017, $\eta_{p}^{2}$ = 0.08), as seen in Figure 1. Post hoc Bonferroni-adjusted simple main effects testing did not reveal between-group differences at any of the delays (*p* > 0.05). The delay by group interaction was no longer statistically significant after adjusting for BMI (F(1.7, 80.8) = 1.698, *p* = 0.193, $\eta_{p}^{2}$ = 0.03).

In a combined participant sample including both individuals with PH and healthy controls, mean office SBP and DBP values were associated with results of the rapid visual processing test (r = −0.431, *p* = 0.001 [SBP*RVPA], r = −0.404, *p* = 0.002 [DBP*RVPA], r = 0.612, *p* < 0.001 [SBP*RVPPFA], r = 0.562, *p* < 0.001 [DBP*RVPPFA]). This finding was also present if central BP measures were considered (r = −0.331, *p* = 0.013 [cSBP*RVPA], r = −0.276, *p* = 0.041 [cDBP*RVPA], r = 0.385, *p* = 0.004, [cSBP*RVPPFA], r = 0.286, *p* = 0.034 [cDBP*RVPPFA]).

Higher DBP during 24 h ABPM was correlated with better performance on the spatial working memory (SWMS) task (r = −0.310, *p* = 0.025 [24-h DBP], r = −0.322, *p* = 0.020 [Daytime DBP], r = −0.291, *p* = 0.036 [Nighttime DBP]). Higher nighttime SBP dipping was associated with better performance in the task of categorical fluency (r = 0.279, *p* = 0.045).

Higher participant BMI was associated with worse results in measures of non-verbal and general intelligence (r = −0.317, *p* = 0.019 [NIQ], r = −0.286, *p* = 0.036 [IQ]), attention and processing speed (r = 0.285, *p* = 0.037 [MTSPS82], r = 0.273, *p* = 0.046 [MTSRCAMD]), visual memory and new learning (r = −0.438, *p* = 0.001 [PALFAMS28], r = 0.369, *p* = 0.006 [PALTEA28]), reaction time (r = 0.413, *p* = 0.002 [RTIFMDMT], r = 0.272, *p* = 0.047 [RTISMDMT]), and sustained attention (r = −0.439, *p* = 0.001 [RVPA], r = 0.280, *p* = 0.041 [RVPMDL], r = 0.399, *p* = 0.003 [RVPPFA]).

**Figure 2:**
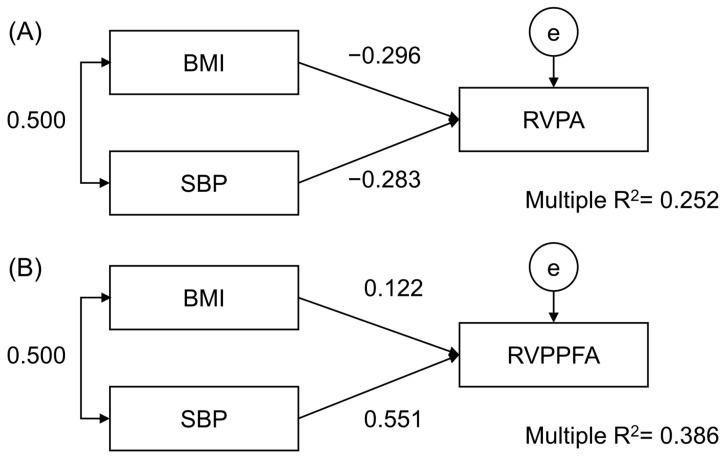
Regression analysis showing the relationship between body mass index (BMI), mean office systolic blood pressure (SBP), and tests of sustained attention: RVPA (**A**) and RVPPFA (**B**). Correlation (two-headed arrow) and standardized regression estimates (single-headed arrows) are shown. e—error.

Regression analysis testing for the association of BMI and SBP with one measure of sustained attention revealed similar weights of both former variables on this cognitive domain (β_SBP_ = −0.001, *p* = 0.038, β_BMI_ = −0.003, *p* = 0.031), explaining 25.2% of its variance (Figure 2A). In an analogical model, only SBP was a statistically significant regression predictor of an alternative measure of the same task (β_SBP_ < 0.001, *p* < 0.001, β_BMI_ < 0.001, *p* = 0.325), explaining 38.6% of its variance (Figure 2B). Low absolute numbers of unstandardized β coefficients emerged from the intrinsic characteristics of the tests employed.

## 4. Discussion

In the study presented, we compared performance between young adults with PH and healthy controls in tasks dedicated to various cognitive domains. Our principal finding was worse performance in tests measuring sustained attention and response speed among individuals with PH. Furthermore, we found partial evidence of differing verbal material forgetting curves in the two groups. If observed across the life spectrum, data supporting cognitive dysfunction in individuals with PH has emerged from studies heterogeneous in both design and outcome measures [8,9,16]. Nevertheless, there is substantial reason arising from cross-sectional and longitudinal neuropsychological studies to regard PH as an important mediator of brain damage both early and late in life. For instance, underperformance in tasks of working memory, constructional skills, verbal memory, and executive functioning has been observed several reports from children and adolescent groups [17,18,19]. A recent systematic review and meta-analysis summarizing studies in adult (age 40–65 years) hypertension reported negative associations between BP in midlife and memory, executive function, attention, global cognition, visuospatial organization, and psychomotor speed in older age [16]. At midlife, the same report suggested no relationship between BP and attention, but a negative relationship between BP and memory, executive function, and global cognition. Therefore, while the presence of PH appears to impact multiple cognitive domains in both very young and middle-aged individuals, the cognitive outcomes that become the most affected vary. To the best of our knowledge, hypertension-associated cognitive dysfunction has been severely understudied among young adults (aged 18 to 45 years) [9]. While poorer performance in tasks of visual recognition, digit symbol, and tapping speed in early adulthood was suggested several decades ago [20], major studies pointing to the importance of cognitive testing in this group of the population have started to emerge only recently. For example, data from the Young Fins study including around 2000 participants suggest that an inverse association between elevated SBP trajectories from childhood and episodic memory, as well as associative learning, exists in adults as young as 42 ± 5 years [21]. However, as reminded by the authors of the report, in the age group of <45 years, only a single modifiable dementia risk factor, low education, was emphasized in the Lancet Commission 2020 report for dementia prevention, intervention, and care [21,22]. As PH starting in early adulthood is known to be associated with greater odds of left ventricular hypertrophy, coronary calcification, albuminuria, and diastolic dysfunction, with the current study we attempted to further acknowledge the brain as another important target organ susceptible to damage from PH [23].

Our findings suggest that the differences in cognitive profiles between young adults with and without PH remain very subtle. Notably, the performance of both groups was comparable in paper-pencil tasks of global cognition, verbal fluency, and short-term verbal memory. During computerized testing, participants with PH were also rated similarly in task of working memory, visual memory, and executive functioning. Therefore, most neuropsychological tools that are recommended for use in older individuals with PH, especially those evaluating global cognition (e.g., the Mini–Mental State Examination or the Montreal Cognitive Assessment), would most likely be prone to miss any slight and selective dysfunction in early adulthood [24]. Furthermore, our study indicates that the cognitive domains affected in young adults with PH differ from those in children and adolescents (predominant impairment in executive functioning) [17] and older adults (memory, executive functioning, and global cognition) [16]. Attention and response speed, which were predominantly affected in the group with PH, have been shown to be at their peak in individuals aged 20–40 years in comparison to other age groups [25,26]. As data from pooled samples in midlife indicate no association between BP and attention [16], such dysfunction may be specific for PH-associated brain damage in young adults [21]. An alternative explanation for this finding could be linked to the intrinsic characteristics of the neuropsychological tests used to assess these domains, such as their higher scale variability and more sensitive units of measurement (e.g., milliseconds required to respond rather than points), leading to improved discrimination between small participant groups [27]. However, this would not account for statistical differences in variables of sustained attention in which variance was low (i.e., RVPA) or performance in both groups were close to a ceiling level (i.e., RVPPFA). We therefore believe that our data justify future exploration of the usefulness of attention and response time as potential markers of BP-associated brain damage.

A noteworthy aspect of our findings was the apparent loss of between-group differences in attention and response speed after adjusting for BMI. While BMI, as a substantial confounder, was independently associated with poorer performance in these tasks, subsequent regression analyses showed that, at least in tests of sustained attention, the role of office SBP also remained statistically significant in explaining the variance of this cognitive domain. Based on systematically reviewed data, obesity is clearly associated with cognitive dysfunction across most domains; however, its specific role as an independent and possibly causative factor of such impairments remains debatable [28,29,30,31]. Our data revealed a synergy between both BMI and SBP that were used as predictors in one of the tests of sustained attention. As only SBP was statistically significantly associated with an alternative variable of the same task, it remains difficult to define the independent effects of BMI and SBP on this domain. Moreover, BMI had wider associations than BP across the spectrum of all cognitive measures used in our study. In summary, current results suggest that, among middle-aged adults, office SBP has an independent relationship with sustained attention, while higher BMI may be linked to lower levels of general intelligence, attention and processing speed, visual memory and new learning, reaction time, as well as sustained attention.

Interestingly, in our study, neither central BP nor 24 h ABPM results had any major associations with cognitive outcomes that would suggest their superiority in comparison to office BP measurements. While the value of central BP in assessing target-organ damage among adults remains hardly defined, results of 24 h ABPM have been shown to better predict such damage as well as other adverse outcomes by providing a more a more reliable reflection of BP values throughout the day and night [7]. In this sense, our results contradict previous studies with older adults that have suggested that 24 h ABPM data, especially nighttime dipping data, are more associated with neuropsychological participant outcome than office BP [32,33,34]. Nighttime dipping was associated only with better verbal fluency in our sample, and higher 24 h DBP values were linked to higher strategy use in the spatial working memory task. The latter findings, however, should be interpreted with discretion, as no statistical differences were found between the PH and control groups in these tasks.

The results of the current study should be interpreted with consideration for its limitations. Firstly, the study sample was relatively small, emerging from a single-center cross-sectional design. This prevented us from conducting in-depth subgroup analyses, based on more specific BP patterns (e.g., defined by ABPM) within the sample and did not provide sufficient power to detect any subtle differences in domains other than response time and sustained attention. Responding to the introduction of potential bias, BMI was recognized as an important confounder and included in sensitivity analyses to differentiate between BP and BMI effects on relevant neuropsychological variables. Furthermore, we could not include reliable data regarding the duration of being exposed to hypertension within our analyses. Finally, we did not adjust our analysis for multiple testing, thus increasing the risk of Type I errors and spurious findings.

## 5. Conclusions

We presented the results of a cross-sectional study comparing cognitive profiles between young adults with PH and their healthy counterparts. While we hypothesized that individuals with PH would perform worse in most cognitive domains, our data suggested such impairment only in tasks of response speed and sustained attention. Higher systolic office BP remained associated with levels of sustained attention, independently of BMI. Future studies with larger sample sizes are necessary to confirm selective dysfunction in response time and sustained attention in young adults with PH, as well as the independence of such dysfunction from BMI and other confounding effects.

## Figures and Tables

**Table 1:** Anthropometric and cardiovascular characteristics of the study sample. BMI—body mass index, cSBP/cDBP/cPP—central blood pressure or pulse pressure measurement, DBP—diastolic blood pressure, MAP—mean arterial pressure, PP—pulse pressure, PPA—pulse pressure amplification, SBP—systolic blood pressure.

Characteristic	Healthy Controls (Median (IQR) or Mean (SD)), *n* = 22	Hypertension Group (Median (IQR) or Mean (SD)), *n* = 33	Independent Sample Test Result and *p* Value
Age, years	36.1 (27.5–39.8)	38.8 (33.2–41.6)	Z = −1.581, *p* = 0.114
Height, cm	177.2 (6.8)	179.6 (8.2)	t = −1.102, *p* = 0.276
Weight, kg	76.3 (12.1)	97.0 (12.4)	t = −6.021, *p* < 0.001
BMI, kg/m^2^	24.2 (3.1)	30.2 (4.7)	t = −5.152, *p* < 0.001
Waist, cm	89.6 (14.6)	103.8 (11.8)	t = −3.831, *p* < 0.001
Hip, cm	99.8 (8.6)	111.9 (10)	t = −4.281, *p* < 0.001
Waist-hip ratio	0.9 (0.8–1.0)	0.9 (0.9–1)	Z = −1.213, *p* = 0.225
Waist-height ratio	0.5 (0.1)	0.6 (0.1)	t = −3.551, *p* = 0.001
SBP, mmHg	117 (108.9–124.1)	131 (122.3–145.3)	Z = −4.038, *p* < 0.001
DBP, mmHg	72.5 (67.4–75.6)	86 (76–96)	Z = −3.833, *p* < 0.001
PP, mmHg	43.6 (6.5)	48.6 (9.3)	t = −2.181, *p* = 0.034
24-h SBP, mmHg	112.9 (6.6)	128.0 (11.9)	t = −5.879, *p* < 0.001
24-h DBP, mmHg	67.5 (65.0–72.5)	79.5 (75.0–86.3)	Z = −4.924, *p* < 0.001
24-h MAP, mmHg	88.7 (4.8)	102.3 (9.6)	t = −6.828, *p* < 0.001
Daytime SBP, mmHg	116.7 (7.8)	130.6 (12.3)	t = −5.015, *p* < 0.001
Daytime DBP, mmHg	71.4 (5.3)	83.2 (9.2)	t = −5.913, *p* < 0.001
Daytime MAP, mmHg	92.2 (5.5)	105.0 (9.9)	t = −5.999, *p* < 0.001
Nighttime SBP, mmHg	102.2 (6.5)	117.7 (11.9)	t = −6.059, *p* < 0.001
Nighttime DBP, mmHg	59.5 (53.5–66.5)	70.5 (66.0–73.5)	Z = −4.324, *p* < 0.001
Nighttime MAP, mmHg	80.0 (73.5–83.0)	91.5 (86.0–95.0)	Z = −4.952, *p* < 0.001
SBP dipping, %	12.3 (5.9)	9.7 (6.0)	t = 1.500, *p* = 0.140
DBP dipping, %	16.3 (8.2)	14.3 (5.8)	t = 1.045, *p* = 0.301
PWV, m/s	6.9 (6.0–7.7)	7.9 (7.2–9.2)	Z = −3.172, *p* = 0.002
cSBP, mmHg	103 (99.8–108.3)	119 (108.5–134.0)	Z = −4.538, *p* < 0.001
cDBP, mmHg	72.5 (67.5–75.8)	81 (73–94)	Z = −3.733, *p* < 0.001
cPP, mmHg	33.0 (27.0–35.3)	38 (32–43)	Z = −2.891, *p* = 0.004
PPA (cPP/PP)	0.7 (0.1)	0.8 (0.2)	t = −1.808, *p* = 0.076

**Table 2:** Comparison of performance between healthy controls and individuals with primary arterial hypertension across different neuropsychological tests. Arb. unit—arbitrary unit, c–complex interpretation, l—lower is better, h—higher is better, IQ—intelligence quotient, MTS—Match to Sample Visual Search, PAL—Paired Associates Learning, RTI—Reaction Time Task, RVP—Rapid Visual Information Processing, SOC—Stockings of Cambridge, SSP—Spatial Span, SWM—Spatial Working Memory, VLS—verbal-logical story task, * *p* < 0.05. For detailed descriptions of separate variables please refer to the section “Cognitive Outcomes” and Appendix A.

Neuropsychological Measure	Healthy Controls (Median (IQR) or Mean (SD)), *n* = 22	Hypertension Group (Median (IQR) or Mean (SD)), *n* = 33	Independent Sample Test Result and *p* Value
Paper-pencil assessment			
Categorical verbal fluency, points ^h^	22.6 (4.1)	22.5 (6.6)	t = 0.095, *p* = 0.925
Phonemic verbal fluency, points ^h^	13.2 (4.1)	11.7 (5.0)	t = 1.192, *p* = 0.239
VLS immediate recall, points ^h^	15.6 (2.4)	15.8 (3.1)	t = −0.223, *p* = 0.825
VLS 30 min recall, points ^h^	14.5 (2.6)	13.4 (3.3)	t = 1.234, *p* = 0.223
VLS 24 h recall, points ^h^	14.0 (2.9)	12.8 (3.6)	t = 1.373, *p* = 0.176
Verbal IQ, points ^h^	117.4 (8.9)	112.7 (12.1)	t = 1.558, *p* = 0.125
Nonverbal IQ, points ^h^	116 (108.5–121.3)	112 (99.5–119.0)	Z = −1.204, *p* = 0.229
IQ, points ^h^	121 (108.5–126.5)	115 (104.5–124.5)	Z = −1.385, *p* = 0.166
**Computerized assessment**			
*Match to Sample Visual Search*			
MTSPS82, ms ^c^	2052.2 (1547.0–2548.8)	2116.3 (1868.2–2507.7)	Z = −0.825, *p* = 0.410
MTSRCAMD, ms ^l^	2033.1 (466.4)	2189.9 (450.9)	t = −1.246, *p* = 0.218
*Paired Associates Learning*			
PALFAMS28, points ^h^	17 (12.0–18.3)	13 (10.5–17.0)	Z = −1.812, *p* = 0.070
PALTEA28, points ^l^	3.5 (1.8–12.3)	10 (3.5–15.0)	Z = −1.747, *p* = 0.081
*Reaction Time Task*			
RTIFMDMT, ms ^l^	251.8 (235.1–274.4)	275 (254.0–319.3)	Z = −2.019, *p* = 0.044 *
RTIFMDRT, ms ^l^	341.8 (321.3–393.6)	361 (345.0–403.3)	Z = −1.822, *p* = 0.068
RTISMDMT, ms ^l^	253.5 (234.3–278.3)	260 (213.5–309.5)	Z = −0.472, *p* = 0.637
RTISMDRT, ms ^l^	304.5 (295.1–330.1)	327 (310.5–356.5)	Z = −2.509, *p* = 0.012 *
*Rapid Visual Information Processing*			
RVPA, arb. unit ^h^	0.929 (0.029)	0.900 (0.052)	t = 2.373, *p* = 0.021 *
RVPMDL, ms ^l^	421.5 (397.6–474.3)	439 (404–466.8)	Z = −1.005, *p* = 0.315
RVPPFA, arb. unit ^l^	0 (0–0.01)	0.01 (0–0.01)	Z = −2.052, *p* = 0.040 *
*Stockings of Cambridge*			
SOCITMD5, ms ^l^	11137 (8361.8–18643)	9825.5 (5990.5–14996)	Z = −1.495, *p* = 0.135
SOCMNM5, points ^l^	5.5 (5–6.8)	5.5 (5–6.5)	Z = −0.202, *p* = 0.840
SOCPSMMT, points ^h^	10 (9–11)	9 (9–11)	Z = −0.747, *p* = 0.455
SOCSTMD5, ms ^l^	0 (0–658.1)	0 (0–235)	Z = −0.442, *p* = 0.658
*Spatial Span*			
SSPFSL, points ^h^	6 (6–7)	7 (6–7.5)	Z = −0.392, *p* = 0.695
*Spatial Working Memory*			
SWMBE4, points ^l^	0 (0–0)	0 (0–0)	Z = −0.477, *p* = 0.634
SWMBE468, points ^l^	4 (0–15)	1 (0–9.5)	Z = −1.238, *p* = 0.216
SWMBE6, points ^l^	0 (0–2.8)	0 (0–2)	Z = −0.622, *p* = 0.534
SWMBE8, points ^l^	3 (0–13)	0 (0–7.5)	Z = −1.118, *p* = 0.264
SWMS, arb. unit ^l^	6.5 (5.8–9)	5 (3–8)	Z = −1.565, *p* = 0.118

## Data Availability

Data supporting the findings are available from the authors upon reasonable request, supported by a data analysis plan.

## Appendix A

**Table A1:** Description of computerized cognitive CANTAB (Cambridge Cognition, Ltd.) measures used in the current study.

Match to Sample Visual Search (MTS)
**Domains:** attention and processing speed. **Description:** One pattern is displayed in the middle of the screen. The participant must select a matching pattern appearing in other boxes on the screen as rapidly as possible. **Variables:** MTSPS82—The difference in mean time between presentation of the response stimulus options and the subject selecting the correct box on their first attempt on the eight-pattern assessment trials compared to the two-pattern assessment trials. Calculated across eight-pattern and two-pattern assessed trials where the first attempt was correct. MTSRCAMD—The median time between presentation of the response stimulus options and the subject selecting the correct box (across all assessed trials).
**Paired Associates Learning (PAL)**
**Domains:** visual memory and new learning. **Description:** The participant selects boxes with a matching pattern based on previously shown information. **Variables:** PALFAMS28—The number of times a subject chose the correct box on their first attempt when recalling the pattern locations. Calculated across assessed trials, omitting 12 box level. PALTEA28—The number of times the subject chose the incorrect box for a stimulus on assessment problems, plus an adjustment for the estimated number of errors they would have made on any problems, attempts and recalls they did not reach. This measure allows to compare performance on errors made across all subjects regardless of those who terminated early versus those completing the final stage of the task.
**Reaction Time Task (RTI)**
**Domains:** motor and mental response speed, movement time, reaction time, response accuracy and impulsivity. **Description:** the participant holds a button at the bottom of the screen and has to react to flashing circles above as soon as possible (release the button and tap the flashing circle). **Variables:** RTIFMDMT—The median time taken for a subject to release the response button and select the target stimulus after it flashed yellow on screen. Calculated across correct, assessed trials in which the stimulus could appear in any one of five locations. Measured in milliseconds. RTIFMDRT—The median duration it took for a subject to release the response button after the presentation of a target stimulus. Calculated across correct, assessed trials in which the stimulus could appear in any one of five locations. Measured in milliseconds. RTISMDMT—The median time taken for a subject to release the response button and select the target stimulus after it flashed yellow on screen. Calculated across correct, assessed trials in which the stimulus could appear in one location only. Measured in milliseconds. RTISMDRT—The median duration it took for a subject to release the response button after the presentation of a target stimulus. Calculated across correct, assessed trials in which the stimulus could appear in one location only. Measured in milliseconds.
**Rapid Visual Information Processing (RVP)**
**Domain:** sustained attention. **Description:** participants have to press a button when they detect a target number sequence within a box of changing digits. **Variables:** RVPA—A’ (A prime) is the signal detection measure of a subject’s sensitivity to the target sequence (string of three numbers), regardless of response tendency (the expected range is 0.00 to 1.00; bad to good). In essence, this metric is a measure of how good the subject is at detecting target sequences. RVPMDL—The median response latency on trials where the subject responded correctly. Calculated across all assessed trials. RVPPFA—The number of sequence presentations that were false alarms divided by the number of sequence presentations that were false alarms plus the number of sequence presentations that were correct rejections (False Alarms ÷ (False Alarms + Correct Rejections))
**Stockings of Cambridge (SOC)**
**Domains:** spatial planning and working memory. **Description:** participants move virtual balls to solve a spatial problem. **Variables:** SOCITMD5—Subjects are encouraged to plan their moves before starting to solve the problems. Initial thinking time is the difference in the time taken to select the first ball for the same problem in the solve compared to follow conditions. This measure provides an indication of the time taken to plan the problem solution for assessed problems with 5 moves, discounting movement time. This score may be 0 if the subject is slower in the follow condition. SOCMNM5—The mean number of moves that the subject required to complete problems (calculated over 5 Moves assessed problems only) SOCPSMMT—The number of assessed problems that the subject successfully completed in the minimum possible number of moves. Calculated over all assessed trials. SOCSTMD5—Calculated as the time between selecting the first ball and completing the problem in the solve conditions minus the corresponding time in the follow condition. This time difference is then divided by the number of moves made in the solve phase. This measure provides an indication of any time taken by the subject to plan or re-plan the problem solution after they have made their first move taking into account their movement time, and the number of moves made. Calculated across all 5 move problems. This score may be 0 if the subject is slower in the follow condition.
**Spatial Span (SSP)**
**Domain:** working memory capacity. **Description:** participants must recall a sequence of boxes changing color. **Variable:** SSPFSL—The longest sequence of boxes successfully recalled by the subject.
**Spatial Working Memory (SWM)**
**Domains:** working memory, strategy use. **Description:** participants must find hidden tokens across a set of colored boxes on the screen. **Variables:** SWMBE4—The number of times a subject revisits a box in which a token has previously been found. Calculated across all trials with 4 tokens only. SWMBE468—The number of times the subject incorrectly revisits a box in which a token has previously been found. Calculated across all assessed four, six and eight token trials. SWMBE6, SWMBE8—The number of times the subject revisits a box in which a token has previously been found. Calculated across all trials with 6 (SWMBE6) or 8 (SWMBE8) tokens only. SWMS—The number of times a subject begins a new search pattern from the same box they started with previously. If they always begin a search from the same starting point, it is inferred that the subject is employing a planned strategy for finding the tokens (a low score indicates high strategy use (1 = they always begin the search from the same box), a high score indicates that they are beginning their searches from many different boxes). Calculated across assessed trials with 6 tokens or more.
